# Mitochondria in innate immunity signaling and its therapeutic implications in autoimmune diseases

**DOI:** 10.3389/fimmu.2023.1160035

**Published:** 2023-04-12

**Authors:** Yuhao Jiao, Zhiyu Yan, Aiming Yang

**Affiliations:** ^1^ Department of Gastroenterology, Peking Union Medical College Hospital, Chinese Academy of Medical Sciences, Beijing, China; ^2^ 4+4 Medical Doctor Program, Peking Union Medical College, Chinese Academy of Medical Sciences, Beijing, China

**Keywords:** mitochondria, innate immunity, immune metabolism, autoimmune disease, therapeutic targets

## Abstract

Autoimmune diseases are characterized by vast alterations in immune responses, but the pathogenesis remains sophisticated and yet to be fully elucidated. Multiple mechanisms regulating cell differentiation, maturation, and death are critical, among which mitochondria-related cellular organelle functions have recently gained accumulating attention. Mitochondria, as a highly preserved organelle in eukaryotes, have crucial roles in the cellular response to both exogenous and endogenous stress beyond their fundamental functions in chemical energy conversion. In this review, we aim to summarize recent findings on the function of mitochondria in the innate immune response and its aberrancy in autoimmune diseases such as rheumatoid arthritis, systemic lupus erythematosus, etc., mainly focusing on its direct impact on cellular metabolism and its machinery on regulating immune response signaling pathways. More importantly, we summarize the status quo of potential therapeutic targets found in the mitochondrial regulation in the setting of autoimmune diseases and wish to shed light on future studies.

## Introduction

Mitochondria are highly preserved double-membrane-bound organelle in eukaryotes with fundamental functions of generating energy *via* oxidative phosphorylation (OXPHOS) (1). Intriguingly, some theories suggest that mitochondria may have evolved from aprokaryotic organism and resided in eukaryotic cytosol symbiotically (2, 3). The double membrane structure of mitochondria, along with their mitochondrial DNA and a highly independent energy supplication system, supports the exogenous origin hypothesis (2, 3), indicating that mitochondria could possibly be involved in the interplay and regulation of immune reactions. This was first confirmed by findings that pointed out the roles mitochondria play in the downstream signaling pathways of multiple pattern-recognition receptors (PRRs) (4). Investigations into mitochondrial apoptosis further suggest their control over the survival and death of vast types of immune cells (5). However, mounting studies have found even more evidence that mitochondria are involved in numerous signaling pathways beyond what was initially expected and that mitochondrial biology is also affected by various cellular signals and metabolites, and *vice versa* (6, 7).

In the study of immune disorders, autoimmune diseases provide an example of their core pathogenesis involves the loss of immune tolerance, the generation of autoantibodies, and host tissue damage (8). At the cellular level, exposure to self-antigen has a complex pathogenic process that eventually leads to the loss of self-tolerance and the abnormal activations of autoreactive T cells and/or B-cells (8).

Innate immune cells, as the first-line defense of the immune system, play a pivotal role in early defense against infectious disease and act as the facilitator for the adaptive immune system (9). Compiling studies have demonstrated the importance of antigen-presenting cells, including dendritic cells (DCs) and macrophages, in sensing danger signals and initiating downstream immune responses (10). Neutrophils and other effector cells, including natural killer (NK) cells and the helper-like innate lymphoid cells (ILCs), also contribute to end-stage tissue damage, exacerbate immune responses, and skew immune responses (11). During this process, PRRs, expressed by innate immune cells, are vital players in detecting invading pathogens and host cell damage (12). PRR recognition of pathogen-associated molecular patterns (PAMPs), expressed by microorganisms, and damage-associated molecular patterns (DMAPs), expressed by host cells, is the key driver of initial innate immune reactions (12). More specifically, sensing PAMPs and DMAPs triggers downstream signaling to recruit additional immune cells, augment cytokine production, and activate adaptive immune responses. Mitochondria get involved in this grand banquet through alterations in immune metabolism, participation in immune signaling transduction, and exacerbation of immune reactions.

Among these comprehensive mechanisms involved in the pathogenesis of autoimmune diseases are numer ous intermediates, such as nucleic acids, key transcription factors, and different immune cell subsets, which are under comprehensive study to unveil potential therapeutic targets. At the organelle level, mitochondria have emerged as critical participants in initiating and progressing multiple autoimmune diseases. And the fact that mitochondria interact with the immune system can give a reasonable explanation of how mitochondria are involved in the pathogenesis of autoimmune diseases.

In this review, we summarize current understandings of potential mechanisms by which mitochondria regulate innate immunity and contribute to the pathogenesis of autoimmune diseases, focus on mitochondrial regulation of immune signaling pathways, immunometabolism, and apoptotic pathways.

## Mitochondria in initiation and transduction of innate immune responses

Innate immune cells recognize pathogenic and damage signals through the expression of PRR family, such as toll-like receptors (TLRs), nucleotide oligomerization domain (NOD)-like receptors (NLRs), retinoic acid-inducible gene-I (RIG-I)-like receptors (RLRs), and C-type lectin receptors (CLRs). Recognition of respective PAMPs and DAMPs by these receptors evokes multiple downstream signals to activate and amplify innate immune responses, eventually upregulating inflammatory mediators for participating in anti-microbial reactions and the pathogenesis of autoimmune diseases under certain conditions (13). Mitochondria are involved in this process by releasing host-derived DAMPs, including phospholipid cardiolipin (pCL), reactive oxygen species (ROS), n-formyl peptides (n-fp), and mitochondrial DNA (mtDNA), when activated by both exogenous and endogenous stress signals (14, 15). And the release of DAMPs recognized by PRRs is a way for mitochondria to participate in triggering downstream innate immune responses (14). Additionally, the transduction of certain PRR-related signaling pathways is also controlled by the mitochondrial platform for signaling molecules (15). The dual role of mitochondria in innate immune responses and the pathogenesis of autoimmune diseases may provide great potential in finding therapeutic targets.

### Mitochondrial ROS

The biochemical generation of mitochondrial ROS (mROS) is an enormously complex process linked to multiple immunological and metabolic events. Approximately 90% of the ROS are biochemical byproducts of OXPHOS at the electron transport chain (ETC) in the inner mitochondrial membrane. Theoretically, electrons from nicotinamide adenine dinucleotide hydrogen (NADH) and flavin adenine dinucleotide (FADH2) are transferred through ETC at complex I-IV. However, leakage of electrons in this process results in the formation of superoxide anion radicals, namely, mROS (16, 17). Other biochemical reactions, such as nicotinamide adenine dinucleotide phosphate (NADPH) oxidase (NOX) complexes and uncoupled nitric oxide synthase reactions at the meantime also generate certain amounts of non-m ROS, especially in phagocytic cells, including macrophages, DCs, and neutrophils (18–20). Studies have shown that the activation of TLRs in innate immune cells sends signals *via* tumor necrosis factor (TNF) receptor-associated factor 6 (TRAF6) and evolutionarily conserved signaling intermediate in toll pathways (ECSIT), leading to the phagocytosis of mitochondria and eventually contributing to mROS production (21). The NLR family, another class of cytoplasmic PRRs, also potentially increases mROS levels upon its activation through downstream regulators, such as NLRP3 inflammasomes and NLRX1 (22).

Notably, mROS is constitutively produced from OXPHOS at the physiological status that supports many signaling pathways and maintains the normal machinery of the innate immune system. Owing to the strong oxidation capability of mROS, it exerts solid activity of anti-microorganisms *via* direct oxidation of microbial proteins and lipids (23). However, mROS is not only limited to its role in facilitating the antibacterial response but also plays a significant role in immune cell differentiation, maturation, and polarization. During the hematopoiesis of immune cells, metabolic control of cell differentiation and death occurs (24). The accumulation of mitochondrial mass and a decreased demand for glycolysis in the later developmental stages lead to an upregulation of OXPHOS and mROS production, which drives the early differentiation of naïve immune cells (25, 26). A single-cell level transcriptome study has also suggested that the potential relationship between ROS and myeloid cell differentiation *via* is built by modulating gene expression related to cellular metabolism (27). As to mature innate immune cells, mROS, more specifically, is crucial for the induction and maintenance of M1 macrophage (the proinflammatory macrophage subset) polarization (28). By activating nuclear factor-kappa B (NF-κB) and p38 mitogen-activated protein kinase (MAPK) signaling pathways, mROS can promote the expression of proinflammatory genes in macrophages, synthesizing numerous factors like TNF-α and interleukin (IL)-6 (29). mROS is also a prerequisite for the activation of nucleotide-binding oligomerization domain (NOD)-like receptor family, pyrin domain containing 3 (NLRP3) inflammasome and the subsequent production of IL-1β and IL-18 *via* initiating caspase-1-dependent cleavage (which is discussed in detail in the following section) (30). Moreover, mROS can directly enhance immune cell function and augment innate immune responses *via* its downstream upregulation of hypoxia-inducible factor-1α (HIF-1α) and NF-κB, leading to the proinflammatory cytokine production and upregulated cytotoxicity (31, 32).

In recent years, there has been growing interest in the role of mROS in the pathogenesis of autoimmune diseases such as systemic lupus erythematosus (SLE), Sjögren’s syndrome (SjS), and rheumatoid arthritis (RA). While mROS has an essential impact in regulating cellular signaling pathways, an overproduction of mROS in response to cellular or mitochondrial stress, represented by hypoxia and inflammation, could be pathogenic (33). The accumulation of mitochondria mass and mROS production is observed in autoimmune diseases such as SLE, whereas the NOX complexes related ROS in phagocytes is deficient, which may predispose to SLE (34). Studies have shown that the increased mROS level is likely to contribute to NETosis (the formation of neutrophil extracellular traps, NETs), ultimately leading to direct tissue damage, the upregulation of interferons (IFN), and clinical progression of lupus (34, 35). In clinical trials, metformin has been studied in individuals with mild to moderate systemic lupus erythematosus (SLE), and it has been reported to reduce disease relapse, potentially by reducing NET formation and IFNα production by plasmacytoid dendritic cells (36). Moreover, ROS production *via* NOX complexes is also found to be downregulated or even absent in SLE, SjS, and RA (37–39). The deficiency of ROS released from NOX complexes might be a compensatory reason why mROS production and NETosis are upregulated in autoimmune diseases. When mitochondrial fission factor Drp1 is depleted *via* Rab4A and mitophagy is diminished, an accumulation of mitochondria mass is observed in SLE patients (40). These findings are consistent with the fact that Rab4A expression is increased in phagocytes in a lupus mouse model, indicating that Drp1 might serve as a potential therapeutic target for SLE (41). mROS is also associated with M2 macrophage (the anti-inflammatory macrophage subset) polarization induced by spermidine (30). Spermidine can markedly increase the level of mitochondrial superoxide and H_2_O_2_ that can activate AMP-activated protein kinase (AMPK), ultimately upregulating HIF-1α, which is needed for the expression of anti-inflammatory genes and induction of autophagy (30). And this phenomenon has been proven in a Dextran Sulfate Sodium (DSS)-induced inflammatory bowel disease (IBD) murine model, which could be ameliorated after treatment with spermidine *in vitro* (30).

Treatments targeting mROS and mitochondrial oxidative stress in SLE and RA have great potential. The antioxidants mitoquinone (mitoQ) and [2-(2,2,6,6-tetramethylpiperidin-1-oxyl-4-ylamino)-2-ox-oethyl] triphenylphosphonium chloride monohydrate (mito-TEMPO) have shown promise in ameliorating SLE *via* inhibition of mROS. Administration with mitoQ can result in the downregulation of mROS releasing, reduced NET formation and NETosis, as well as decreased type I IFN production *via* inhibition of oligomerization of mitochondrial antiviral signaling protein (MAVS), in both patient and murine models (34, 42, 43). Other novel compounds, such as polydatin, that is extracted from a traditional Chinese herb *Polygonum cuspidatum*, are also proven to exert therapeutic potential in SLE *via* by restraining ROS and NETosis-mediated damage (44). In RA, resveratrol could alleviate disease progression *via* its inhibition of ROS and MAPK signaling, eventually resulting in suppressed angiogenesis (45). Celastrol is also found to inhibit RA *via* its downregulation of NF-κB-NLRP3 inflammasome axis *via* ROS (46). These findings verify that mROS and oxidative stress may be promising targets for developing novel therapies for autoimmune diseases, especially for SLE and RA. But further research is also needed to deeply and fully understand how mROS elaborately works in the pathogenesis of these diseases and to develop effective treatments targeting this link upon mechanism.

### Mitochondrial DNA

mtDNA is a circular double-strand molecule composed of 37 genes that encode 13 mitochondrial-specific proteins for OXPHOS, 2 ribosomal RNA and 22 transfer RNA (47). All these molecules are pivotal for cellular metabolism, calcium homeostasis, and programmed cell death (48). However, upon cell death processes such as apoptosis and necrosis or mitochondrial damage, the integrity of mitochondria is ruptured and mtDNA is released into either cytosol or extracellular matrix. In contrast to self-DNA in the nucleus, mtDNA is considered a foreign substance due to its distinct methylation status (49, 50). Therefore, exposure to mtDNA sends a danger signal to innate immune sensors and triggers downstream immune responses, consequently contributing to the pathogenesis of certain autoimmune diseases. For example, mtDNA has been detected in the synovial fluid and serum of RA patients, where it can activate immune cells such as phagocytes and overproduce proinflammatory cytokines, leading to joint inflammation (51). In patients with SLE, abundant mtDNA released from damaged cells emerges in the circulating and can activate the type I IFN pathway *via* the endosomal TLR9 (52). Moreover, emerging evidence suggests that free circulating mtDNA is present in a range of inflammatory diseases, including autoimmune diseases, infectious diseases, cardiovascular diseases, and trauma, indicating their highly preserved relationship with inflammation (53). In a groundbreaking study, injecting mtDNA into joints in mice has been shown to induce sterile inflammation and arthritis (54). In conclusion, the role of mtDNA in immune responses has significant implications for understanding and treating inflammatory diseases. Meanwhile, with regards to SLE, there is increasing attention on mtDNA instability as a novel antigen whose antibodies have shown a positive correlation with anti-dsDNA, which is also observed in RA, another prototype of autoimmune diseases (55, 56).

As atype of PRR, TLRs can be activated by specific ligands to recognize various targets, covering bacterial and mtDNA, and evoke immune responses through the activation and nuclear localization of NF-κB *via* p38 and MAPK pathways (57). In humans, 10 types of TLR types have been identified. And the characteristics of ligands for TLR9 are hypomethylated CpG motifs, which are common between bacterial DNA and mtDNA (58, 59). TLRs are widely expressed in various immune cells, including monocytes, macrophages, and plasmacytoid DCs, and are located on the endosomal membrane of these cells. After the formation of the DNA-bound TLR9 complex, myeloid differentiation primary response 88 (MyD88) would be recruited to furtherly induce inflammatory signaling (48). Abnormally released mtDNA into the circulation or mtDNA-associated NETs are capable of instigating drastic immune responses through the above mechanism not only in systemic inflammatory response syndromes (SIRS) but also in hematological malignancies, and neutrophils are thought to play an essential role in this process (60, 61). These innate immune signaling pathways play a crucial role in the development and progression of non-alcoholic steatohepatitis (NASH), mainly involving hepatic stellate cells and macrophage-like Kupffer cells. In a study comparing TLR9 knockout (TLR9^-/-^) mice and wild-type mice on the same choline-deficient amino acid-defined diet, the wild- type mice were found to bemore prone to develop NASH (62). Meanwhile, the levels of oxidated mtDNA in hepatocytes and plasma of both mice and humans with NASH are immensely elevated, indicating that mitochondrial damage is associated with the development of NASH in a TLR-dependent manner. And strikingly, treatment with a TLR9 antagonist treatment has been found to be effective in treating NASH, suggesting that targeting TLR9 signaling may be a promising therapeutic strategy for this disease (63). In parallel, the pivotal interactions have also contributed to inflammation and autoimmunity that correlate with the pathogenesis of SLE (64). In SLE, neutrophils abundant in oxidized mtDNA could activate TLR7 upon the presence of type I IFN and anti-Sm/ribonucleotide protein (RNP) autoantibodies. TLR7 activation can block the phosphorylation of transcription factor A mitochondrial (TFAM), which leads to inefficient disassembly of oxidized mtDNA from TFAM (65). Consequently, oxidized mtDNA accumulates within mitochondria instead of being directed to lysosomes for degradation and is extruded as endogenous foreign substances, evoking formidable inflammation (65). In RA, while limited research has been conducted on mtDNA-induced innate immune responses through TLRs, research has focused more on the adaptive immune system, particularly T cells (56, 66).

Recent studies have shed light on another mechanism by which cytosolic DNA, including mtDNA, triggers inflammatory responses. Upon recognition of mtDNA, cyclic GMP-AMP synthase (cGAS) undergoes a conformational change that facilitates the conversion of ATP and GTP into 2’3’-cyclic GMP-AMP (cGAMP) (67). Subsequently, cGAMP causes conformational changes in the C-terminal tail of STING, which leads to its translocation from the endoplasmic reticulum to the Golgi apparatus. And this results in the recruitment and activation of Tank binding kinase 1 (TBK1) and IKB kinase (IKK), which phosphorylate and activate the transcription factor interferon regulatory factor 3 (IRF3) and NF-κB, resulting in an evoked inflammatory responses featured by type I IFN (68–70). And this pivotal mechanism has been found to contribute to the occurrence and development of systemic inflammatory diseases by numerous studies. As is well known, RA is one of the classic TNF-driven diseases characterized by an IFN signature in affected tissues (71, 72). It has been found that interactions between mtDNA and cGAS contribute to TNF-induced IFN production, which in turn contributes to the joint pathology of RA. In the presence of TNF, PINK1-mediated mitochondrial mitophagy in macrophages is inhibited, leading to an increase in cytosolic mtDNA levels, subsequently inducing and amplifying the inflammatory responses described above. Arthritis-related indicators, such as inflammatory cell infiltration and joint swelling, are significantly ameliorated in the K/BxN arthritis model with cGAS^-/-^ compared to the wild type (73). Additionally, DNase II, an important catalytic enzyme capable of digesting chromosomal DNA and nuclei from apoptotic cells to maintain host homeostasis, has also been implicated in autoinflammation and arthritis when self-DNA degradation fails due to DNase II deficiency. This has been shown to correlate with increased type I IFN in humans and mice *via* sustained cGAS-STING stimulation (74, 75). These findings emphasize the crucial role of mtDNA and its recognition in the development of inflammatory responses and associated diseases.

Inflammasomes are multiprotein complexes consisting of three major components: a receptor, an adaptor, and an effector protein. The receptor protein is a member of the NLR family that recognizes PAMPS or DAMPs, which allows the inflammasome activation. The different types of inflammasomes are mainly distinguished by the molecular receptors they contain, with NLRP3 being one of the most well-studied examples (76). The recognition triggers the oligomerization of the receptor protein and then recruits the adaptor protein, defined as an apoptosis-associated speck-like protein containing a caspase recruitment domain (ASC). ASC acts as a bridging molecule, with its N-terminal pyrin domain (PYD) binding to the receptor protein and its C-terminal caspase recruitment domain (CARD) interacting with the effector protein. Pro-caspase-1, as the effector molecule, undergoes autoactivation, eventually leading to the cleavage and activation of proinflammatory cytokines, such as IL-1β and IL-18, completing the circular process from cell injury recognition to the induction of inflammatory responses (77–80). Although the precise events during the inflammasome activation process are still unclear, it is now evident that mtDNA may rouse the NLRP3 inflammasome pathway by serving as a danger signal or a DAMP. Upon mitochondrial damage or dysfunction, mtDNA is released where it can directly interact with NLRP3, inducing NLRP3 oligomerization and inflammasome activation (79, 81). Another proposed mechanism is that mtDNA can also contribute to NLRP3 inflammasome activation in an mROS-dependent manner (82). mtDNA can stimulate mROS production by binding to the MAVS and initiating the downstream signaling cascades (83). The mROS then activates the NLRP3 inflammasome by inducing mitochondrial damage, potassium efflux, and lysosomal destabilization (77). Furthermore, recent studies have shown that oxidized mtDNA, which is generated by mtDNA damage caused by mROS or other cellular stressors, has a stronger ability to activate the NLRP3 inflammasome than non-oxidized mtDNA (84, 85).

The pro-inflammatory potential of mtDNA highlights the importance of self-clearance to limit inflammatory priming and storm. Multiple intrinsic mechanisms have been identified for mtDNA clearance, including autophagy, phagocytosis mainly mediate *via* by macrophages and DNase-associated degradation (86, 87). Dysfunction in mtDNA clearance has been linked to the pathogenesis of various autoimmune diseases. As aforementioned, Upregulated levels of circulating mtDNA are observed in patients with SLE and RA, which is closely related to the proinflammatory cytokine production and disease progression (51, 52). Defective in mtDNA clearance is also found in IBDs, resulting in the accumulation of mtDNA in the cytoplasm of intestinal epithelial cells and inducing inflammasome activation (88–90). In turn, both autophagic elimination of damaged mitochondria and pharmacological inhibition of mtDNA synthesis effectively prevent inflammasome activation and improve conditions (91, 92). Therefore, strategies targeting mtDNA clearance pathways, such as autophagy and DNase, may hold therapeutic potential for treating autoimmune diseases and warrant deeper investigations.

### Phospholipid cardiolipin

Cardiolipin is a unique phospholipid that is a pivotal structural constituent of mitochondria and is also widely found in bacteria, yeast, and plants. It is characterized by a dimeric phospholipid linked by a glycerol moiety (93). In eukaryotes, cardiolipin presents as an unsaturated form and is strictly located in the inner and outer leaflets of the inner mitochondria membrane. Beyond maintaining mitochondrial membrane integrity and regulating mitochondrial dynamics, abundant studies have revealed that cardiolipin has multiple functions in mitochondrial metabolism, apoptosis, and mitophagy *via* interacting with multiple molecules.

Furthermore, cardiolipin plays a critical role in innate immune responses, particularly under pathological conditions associated with mitochondrial dysfunction, stress, or damage. These abnormal conditions can lead to changes in the level of cardiolipin saturation and oxidation, resulting in the translocation of cardiolipin to the cytosolic side of the outer mitochondrial membrane (94). Once externalized, cardiolipin would act as a DAMP molecule to activate the innate immune system through various mechanisms, and one of them involves the direct activation of TLR4 by cardiolipin. In contrast to the unsaturated form that is unique to physiological conditions, saturated cardiolipin can mimic bacterial lipopolysaccharides and bind to MD2 co-receptor of TLR4, leading to cytokine production, such as TNF-α, IP-10, and IL-1β, and fostering a pro-inflammatory environment (95). Another mechanism involves the activation of the NLRP3 inflammasome. Exposed cardiolipin outside the inner mitochondrial membrane can interact with concomitant pro-caspase-1 and NLRP3 associated with mitochondria to induce inflammasome activation and produce IL-1β and IL-18 (95).

The relationship between cardiolipin and the onset of autoimmune diseases largely relies on the presence of anti-phospholipid autoantibodies, which are considered a hallmark of SLE and anti-phospholipid syndrome (APS). These autoantibodies lead to the formation of immune complexes in multiple tissues and contribute to inflammation and tissue damage. However, the actual role of cardiolipin itself is intensely complicated in the setting of inflammation and autoimmune diseases. Sam50, a mitochondrial outer membrane protein, cooperates with Mic60 to bind to cardiolipin, vital for maintaining mitochondrial membrane integrity. Depletion of Sam50 predisposes cardiolipin to externalization and structural remodeling of the inner and outer mitochondrial membranes, ultimately resulting in liver inflammation in mice models (95). Intriguingly, cardiolipin externalization is also a signal for mtDNA release, which activates the cGAS-STING pathway and induces the downstream responses. And the exogenous expression of Sam50 can reverse it and attenuate liver injury (95). Overall, cardiolipin’s role in autoimmune diseases is complex and multifaceted. Future research is urgently needed to help people fully understand the mechanisms involved and how they may be targeted for therapeutic purposes.

### Transduction of immune signaling pathways

The involvement of mitochondria in innate immunity extends beyond the generation of DAMPs such as mROS, mtDNA, and cardiolipin, as discussed above. Mitochondria also act as a transfer station for TLR, NLR, and RLR-related signaling pathways, serving as a bridge between immunoreactive agents and downstream signaling pathways. Through these pathways, mitochondria play a crucial role in regulating the immune response to infection and tissue damage.

Upon activation, TLR initiates downstream signaling transduction that ultimately leads to the nuclear translocation of NF-kB and upregulation of proinflammatory cytokines, particularly type I IFN (96, 97). Mitochondria play a dual role in TLR signaling, acting as both initiators and enhancers of TLR signaling *via* through the release of mtDNA and mROS, as well as key intermediates in TLR signaling transduction. Studies have demonstrated that mitochondria and their certain intermediates are closely related to TLR signaling. For example, TRAF6 acts as an intermediate that connects activated TLR with the ubiquitination of ECSIT in mitochondria, which further contributes to an elevated level of mROS, enhancing the anti-microbial effect and potentially favoring the pathogenesis of autoimmune diseases (4, 98). Additionally, TLR activation could also alter the mitochondria function and *vice versa*. Upregulation of mtDNA is observed upon activation of TLR2 and TLR4, potentially contributing to the induction of local or systemic inflammation (99, 100). Inappropriate activation of TLRs could possibly be linked with autoimmune diseases. Multiple studies have found that the TLR7 and TLR9 signaling pathways could contribute to type I IFN release in SLE (101, 102). In a study of systemic sclerosis, the production of IL-10 by dendritic cells, which induce T helper (Th) 2 immune responses and exacerbate disease progression, is found to be mediated by TLR2 and TLR4 (103).

The NLR family of PRRs is located in the cytoplasm and forms a multi-protein complex known as the inflammasome. Innate immune cells, including dendritic cells, macrophages, monocytes, and neutrophils, are the major source for NLRP3 and NLRX1 inflammasomes to carry out their anti-microbial function (104). However, inappropriate activation of inflammasomes, including NLRP3 and NLRX1, is closely related to multiple autoimmune diseases, such as SLE, RA, and other inflammatory diseases, which is well summarized and discussed in two recently published review articles (105, 106). The priming and activation of NLRP3 and NLRX1 inflammasomes are closely related to mitochondria. Once an immunostimulatory signal transduces through the TLR- MyD88- NF-κB axis, NLRP3 and pro-IL-1β are upregulated for further assembly of NLRP3 inflammasomes (107). The process of NLRP3 activation is also intertwined with several mitochondrial molecules, such as mtDNA and cardiolipin. Oxidized mtDNA can activate NLRP3 and contribute to downstream upregulation of proinflammatory cytokines and activation of immune responses (108). Additionally, cardiolipin can directly bind to the NLRP3 and activate the inflammasome (109). Moreover, if the cardiolipin synthesis pathway is disrupted, it leads to compromised activation of the NLRP3 inflammasome (109). And introducing cardiolipin (excluding other phospholipids) into a damaged cellular system could prompt caspase-1 activation (109). Abnormalities of the NLR family are now recognized as a critical factor invarious inflammatory diseases. Human NLRP1 polymorphisms are correlated with an increased risk of SLE (110), vitiligo (111), and type 1 diabetes (112). In these disease models, a shared pattern of IL-1α, IL-1β, and IL-18 overproduction is observed, which may be the reason for how NLRP1 activation contributes to the pathogenesis of these inflammatory diseases.

The RLR family, comprising of RIG-I, melanoma differentiation-associated gene 5 (MDA5), and laboratory of genetics and physiology 2 (LGP2) plays a critical role in the innate response to viral infections and the production of type I IFN (113). MAVS is activated upon viral RNA recognition by RIG-I or MDA5 and further recruits multiple molecules including TRAF5 and TRAF6 for downstream activation of NF-κB during a typical response to viral infection (114). MAVS, therefore, acts as a central hub in the mitochondrial antiviral response pathway, mediating the interaction between the RLR family of PRRs and downstream effectors. And mutations or dysregulation of MAVS have been implicated in a variety of diseases, including autoimmune disorders. In SLE patients, an abnormal aggregation of MAVS in a prion-like pattern is observed and could contribute to the release of type I IFN, potentially exacerbating the onset of SLE (115). In non-synonymous single nucleotide polymorphisms (SNPs) screening analysis identifies a loss-of-function variant of MAVS is associated with an uncommon subset of SLE patients and correlates with lowered expression of type I IFN (116). Intriguingly, a recent study has shown that MAVS is critical in promoting B cell activation besides their impact on promoting type I IFN production (117). Meanwhile, aberrant RLR activation has also been proven to be associated with the pathogenesis of various autoimmune or inflammatory diseases such as SLE (118), type 1 diabetes (119), and dermatomyositis (120), due to their ability to induce proinflammatory cytokine production and autoantibody generation (121). MDA5 is the autoantigen that induces the production of the anti-MDA5 autoantibody, which directly contributes to the progression of dermatomyositis and significantly increases the risk of interstitial lung disease in these patients (120). RLR mutations or polymorphisms, such as MDA5, are also observed in SLE and dermatomyositis, leading to an increase in the type I IFN signature (122, 123).

NLRX1 is another critical member of the NLR family that also regulates mitochondrial antiviral signaling and might serve as a link between NLR and RLR pathways. It has been shown to negatively regulate the RLR pathway by inhibiting the MAVS and downstream activation of IRF3 and NF-κB (124). This leads to the suppression of the type I IFN and pro-inflammatory cytokine production, which is important for preventing excessive inflammation and tissue damage during viral infections (22). Additionally, NLRX1 has been implicated in the regulation of mitochondrial homeostasis, including mitochondrial fission and fusion, ROS production, and mitophagy (22). Studies have shown the association between NLRX1 and autoimmune diseases such as SLE, RA, and IBD. In SLE, as aforementioned, the aggregation of MAVS may contribute to the elevation of type I IFN and augment autoimmunity (116). However, although NLRX1 was expressed, its level was not affected by the aggregation of MAVS (116). In IBD, NLRX1 was found to inhibit the development of the disease, and its agonist NX13 could increase OXPHOS levels and prevent the progression of IBD (125, 126). Overall, research on NLRX1 in the field of autoimmunity is rapidly developing, yet its exact mechanisms in autoimmune diseases remain to be fully elucidated.

Chloroquine or hydroxychloroquine, the anti-malarial drug, has long been recognized as critical in the treatment of SLE and RA (127, 128). These drugs work by blocking TLR7, 8, and 9, which are involved in autoimmune disease progression (129). However, clinicians must also manage side effects including myopathy, retinopathy, and gastrointestinal symptoms, which may be due to their off-target effects (130). Hence, specific inhibitors targeting TLRs, NLRs, and RLRs could have even more tremendous potential in developing novel treatments for autoimmune diseases. Small molecule inhibitors and other antibodies, nanoparticles, and oligonucleotides that directly inhibit TLRs, NLRs, RLRs, or intermediates in their signaling pathways are now under comprehensive study, referring to a recently published review (121).

## Metabolic pathway

There are six major cellular metabolic pathways that maintain cellular homeostasis: glycolysis, the tricarboxylic acid (TCA) cycle, the pentose phosphate pathway, fatty acid oxidation (FAO) and synthesis, as well as amino acid metabolism. Mitochondria is the site where the TCA cycle and FAO take place. Owing to the diverse functions of different cell types, the metabolic pattern can differ. In various innate immune cells, research has demonstrated abnormalities in mitochondrial metabolism and their association with the development of autoimmune diseases.

### The pyruvate oxidation in innate immune cells

Mitochondria are the center for pyruvate oxidation, facilitated by the enzyme pyruvate dehydrogenase (PDH) that converts pyruvate to acetyl-coenzyme A (CoA) in the mitochondrial matrix, enabling carbon flow into the TCA cycle and maximizing energy extraction from glycolysis. PDH can be inactivated through phosphorylation by pyruvate dehydrogenase kinase (PDK), resulting in lower catalytic activity. In contrast, dephosphorylation of PDH *via* by pyruvate dehydrogenase phosphatase (PDP) activates it. Therefore, PDK and PDP orchestrate the metabolic state within innate immune cells, ultimately affecting the balance between proinflammatory and immunosuppressive functions (131).

Macrophage polarization towards the M1 type is a critical factor in the pathogenesis of several autoimmune diseases. Pyruvate oxidation is associated with enhanced inflammation status in certain situations and PDH activity is directly linked to the polarization of macrophages towards M1 type. Studies using mouse models have verified that inhibiting PDK2 and PDK4, either through genetic deletion or pharmacological inhibition, can entirely prevent this polarization (132). This occurs through enhancing mitochondrial respiration and metabolite rewiring from glycolysis and the TCA cycle. Interestingly, transplantation of PDK2/4-deficient bone marrow into irradiated wild-type mice can alleviate inflammation and reduce obesity-associated insulin resistance. PDK inhibitors, such as sodium dichloroacetate (DCA), have the potential to treat inflammatory diseases, including colitis and experimental autoimmune encephalomyelitis (EAE) (133).

In turn, M1 macrophages exhibit a reduced α-ketoglutarate/succinate ratio, leading to pseudo-hypoxia gloss and an increased stability of HIF-1α, which further PDK1 expression and favors PDH phosphorylated. As a result, the carbon flux into the TCA cycle is blocked at pyruvate oxidation, while the concomitant, represented by lactate, is increased (134–136). In addition, the activity of PDH is also downregulated by nitro oxide (NO) by directly affecting its PDH-E3 *via* S-nitrosation, which is independent of HIF-1α (137). Moreover, inducible nitric oxide synthase (iNOS or Nos2) continuously produces abundant NO to support this process in M1 macrophages (138). Similarly, DCs exhibit increased expression of Nos2 and NO production, indicating their glycolytic commitment to Warburg metabolism (139).

Inhibition of PDH *via* pharmacological strategies including using melatonin was shown to be able to suppress the proinflammatory polarization and associated with relieved clinical outcomes of chronic inflammatory diseases, represented by multiple sclerosis (133, 140).

### The TCA cycle and the electron transport chain in innate immune cells

In the mitochondrial matrix, pyruvate or fatty acid-derived acetyl-CoA combines with oxaloacetate, a four-carbon compound, to form citrate and release CoA. Through a series of eight-step reactions, the TCA cycle regenerates oxaloacetate to generate a complete loop, producing energy and small molecules for anabolism NADH and FADH2 are representative molecules that can yield ATP *via* the ETC, consisting of five multiprotein complexes on the inner mitochondrial membrane. Citrate can also act as a carrier for acetyl-CoA, crossing the mitochondrial membrane and being hydrolyzed by ATP-citrate lyase to generate cytosolic acetyl-CoA, which participates in epigenetic modification (141, 142). The TCA cycle plays a crucial role in maintaining normal cellular functions, including those of innate immune cells. Studies have suggested potential roles for the TCA cycle in regulating immune functions and disease pathogenesis.

From the perspective of energy supply, the TCA cycle is the primary source of long-term activation and energetic supplies for most quiescent or non-proliferative cells, such as M2 macrophages, due to its highly efficient modes of ATP generation. Nevertheless, the metabolic requirements of M1 macrophages undergoing rapid activation necessitate a shift toward glycolysis, even in the absence of hypoxia (143). And glycolysis in this scenario is essential for generating ATP and NADH, as well as providing biosynthetic intermediates for nucleotides, amino acids, and others (143). It is noteworthy that M2 macrophages exhibit an increased mitochondrial oxygen-consumption rate and spare respiratory capacity, in contrast to M1 macrophages.

Although both activated DCs and M1 macrophages primarily rely on glycolysis as their metabolic pathway, their objectives differ. While M1 macrophages use glycolysis to generate additional fuel, DCs prioritize the *de novo* synthesis of fatty acids from glycolysis metabolites to support the synthesis, transport, and secretion of proteins necessary for DCs activation. This is because DCs are non-proliferative cells (144). Additionally, glucose uptake increases at the early stages of DC activation, but as time progresses, OXPHOS becomes the dominant energy source required (145).

### The fatty acid oxidation in innate immune cells

After being activated by esterification with CoA in the cytosol, fatty acids are transported into the mitochondrion through conjugation to carnitine *via* carnitine palmitoyl-transferase (CPT) 1 located at the outer mitochondrial membrane, which is also the rate-limiting step in the pathway. Once inside the mitochondrion, fatty acids are converted back to the activated form by CPT2 and undergo cyclic β-oxidation, resulting in the continuous degradation of fatty acyl-CoA thioesters and yielding numerous acetyl-CoA, NADH, and FADH2. Interestingly, this metabolic pathway has been found to regulate immune functions and support the cellular longevity of macrophages (143).

In contrast to M1 macrophages, where glycolytic metabolism is the predominant way to satisfy their needs in energy and materials, FAO plays an essential role in M2 macrophages, especially for optimal activation, under the drive by signal transducer and activator of transcription 6 (STAT6) and peroxisome proliferator-activated receptor (PPAR) γ coactivator-1β (PGC-1β) (146, 147). Additionally, this pathway is also important to fuel mitochondrial OXPHOS.

Impaired FAO in M1 macrophages can result in the aberrant degeneration and accumulation of fatty acids and their derivative lipoproteins, leading to the production of proinflammatory cytokines, including IL-1α, which can contribute to the occurrence and development of inflammatory pathologies (148). In contrast, the main role of FAO in M2 macrophages is to maintain sustained induction and prolonged survival of the anti-inflammatory subtype at later stages, rather than during early differentiation (149). Furthermore, inducing increased FAO with didymin, which upregulates Hadhb expression, can even convert polarized M1 macrophage into the M2 type, attenuating acute and chronic colitis in mice models (150).

Similarly, macrophages expressing the permanently active mutant form of CPT1A, CPT1AM, exhibit enhanced levels of FAO, leading to lower triglyceride content and reduced production of proinflammatory cytokines, represented by TNF-α and IL-1β, while the expression of anti-inflammatory cytokines remains un affected (151). Overexpression of PGC-1β can also weaken M1 macrophage activation in response to stimuli, such as saturated fatty acids (152). Conversely, inhibition of CPT1A with etomoxir suppresses the FAO commitment and spare respiratory capacity of M2 macrophages which in turn limits their activation in response to IL-4 (153).

The immune functions of DCs are also regulated by the status of FAO. Solid evidence has demonstrated that the expression of the CPT1A gene, whose translation product is the rate-limiting enzyme for β-oxidation, is increased in tolerogenic DCs, leading to the active state of OXPHOS. Inhibition of FAO by etomoxir significantly reduces the secretory levels of tolerogenic cytokines IL-10 and IL-27 whereas proinflammatory cytokines IL-12 and IL-6 remain stable. Moreover, compromised tolerogenic DCs under treatment with dual KC7F2, a selective HIF-1α translation inhibitor, and etomoxir perturb the polarization of naïve CD4+ Th cells towards Th1 (145). Additionally, in DCs, upregulation of PPARα induced by tumor-derived exosomes promotes FAO activity and shifts the predominant pattern from glycolysis toward OXPHOS, resulting in a dysfunctional state. Importantly, the PPARα inhibitor, GW6471, can abrogate this suppressive effect and restore the function of DCs, enhancing IFN-γ producing ability of T cells to promote inflammation (154).

Apolipoprotein C-III (Apoc3) is a component of triglyceride-rich lipoproteins that is known to stimulate monocytes in an NLRP3 inflammasome-dependent manner, resulting in pro-inflammatory activity (155). In addition, a study on Apoc3-transgenic mice, which are characterized by high levels of plasma triglycerides and free fatty acids, showed an imbalance in the energy supply of NK cells, with increased FAO and decreased glycolysis (155). This led to abnormal activation of the PPAR-γ signaling pathway and downstream signaling through PTEN-AKT-mTOR/FOXO1. The changes also severely impaired the number and function of NK cells in multiple dimensions, including decreased capacity for proliferation, IFN-γ production and secretion, and degranulation against pathogenic targets. However, the impaired state can be partially reversed by administering PPAR-γ inhibitors such as GW9662 or CTP1α inhibitors like etomoxir (155).

## Metabolites

Mitochondrial metabolites produced by innate immune cells undergoing metabolic reprogramming also own powerful capacity to precisely influence the direction of immune responses and modulate actual functions for adopting the surrounding environment in a macroscopic perspective rather than leading to nuanced or specific responses to stimuli (131). Perturbations to metabolites can have significant impacts on inflammatory signaling, epigenomic regulation, and other cellular functions.

Macrophage subtypes exhibit distinct differences in their TCA cycle patterns. M2 macrophages have an intact TCA cycle that is closely connected to the ETC, providing a steady energy supply while avoiding excessive mROS production. The TCA cycle generates Uridine diphosphate N-acetylglucosamine (UDP-GlcNAc) intermediates essential for N-glycosylation, including for the lectin/mannose receptor, which is critical for the normal immune function of M2 macrophages. Inhibitors of N-glycosylation significantly decreased the expression of CD206 and CD301, notable M2 markers, indicating that M2 activation and polarization were prevented (156). The transcription factor c-Maf plays a crucial role in this process (157). In contrast, in M1 macrophages, the TCA cycle is “broken” at two sites where citrate and succinate are converted to isocitrate and fumarate, respectively (15). Fumarate forms a variant of a pathway called the aspartate-arginosuccinate shunt. These changes not only alter the energy supply pattern of M1 macrophages but also lead to the accumulation of by-products that maintain a high-intensity pro-inflammatory status, such as malates (156).

### Citrate

In the context of metabolic reprogramming, citrate, an important TCA cycle metabolite, is exported from the mitochondria *via* the mitochondrial citrate carrier and utilized for producing mediators with proinflammatory potentials (15). Citrate-derived lipogenesis supports membrane biogenesis, which is essential for cell differentiation and proliferation but also can be a source of arachidonic acid needed for prostaglandin synthesis. And this phenomenon has been observed in activated DCs, where citrate is used to enhance antigen presentation efficiency. Additionally, citrate can be shunted into synthetic pathways of nitric oxide through two steps, NADPH is first produced *via* malic enzyme and pyruvate and ultimately converted to NO by iNOS. At the same time, NAPDH can also be furtherly oxidated to produce ROS (143). In M2 macrophages, however, there is limited NO due to increased expression of arginase, leading to a low concentration of arginine (158). In contrast, M1 macrophages utilize immune response gene 1 protein to decarboxylate citrate-derived cis-aconitate to itaconate. This metabolic product possesses direct antimicrobial effects and inhibits succinate dehydrogenase, resulting in massive accumulation of succinate due to failed conversion to fumarate and regulating ROS production (159–161).

### Succinate

Succinate significantly stabilizes the transcription factor HIF-1α, which is vital for the sustained production of IL-1β, under normoxia by inhibiting the activity of prolyl hydroxylases, resulting in enhancing the inflammation responses (134). In addition, succinate can also inhibit PDH to limit acetyl-CoA and restrict acetyl-CoA from entering the TCA cycle, forming a vicious cycle together with the aforementioned commitments (162). However, the HIF-1α expression in the tolerogenic DCs is more stabilized and coordinated by increased AKT and mTORC1, responsible for the upregulation of several glycolytic enzymes to promote this metabolism. KC7F2, a HIF-1α inhibitor, significantly reduces the percentage of HIF-1α positive tolerogenic DCs and suppresses the secretion of tolerogenic cytokines, without affecting the production of proinflammatory cytokines (145).

### Glutamine-derived α‐Ketoglutarate

Glutamine is a critical source of α‐ketoglutarate, which serves as a vital intermediate in the TCA cycle and plays a role in controlling innate immune responses (131). Glutamine is converted to glutamate *via* glutaminase, which is further dehydrogenated into α‐ketoglutarate *via* glutamate dehydrogenases (131). Studies have found an increased glutamine consumption and upregulated glutaminase activity in immune cells upon their stimulation, indicating the role of glutamine in immune cell proliferation (163). In macrophages, α‐ketoglutarate could drive the polarization of macrophages *via* modulating marker genes of M1 and M2 types to exert their anti-inflammatory effect (164). α‐ketoglutarate could inhibit the production of proinflammatory cytokines such as IL-6 and IL-12 and also the expression of IL-1β, IL-6, and TNF-α in macrophages possible *via* the induction of Arg1 and Mrc1 (165). Supplement with dimethyl-α‐ketoglutarate, a cell membrane-permeable analog of α‐ketoglutarate, could attenuate inflammation *via* driving M2 polarization and inhibition of proinflammatory cytokine production (165, 166). Treatment with melatonin, which could induce α‐ketoglutarate production, could promote M2 polarization and potentially prevent inflammatory diseases (167).

## Concluding remarks

**Figure 1:**
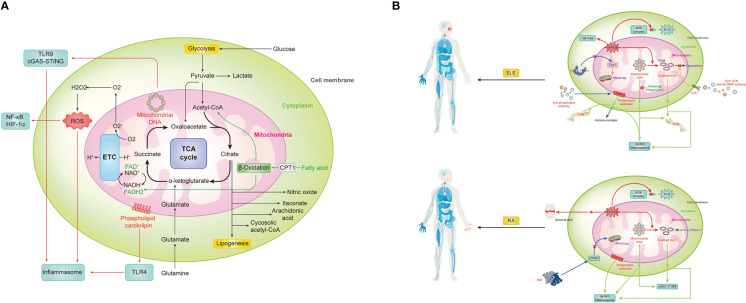
Mitochondrial in innate immunity and aberrant status for autoimmune diseases, represented by two prototypes, SLE and RA. **(A)** Mitochondria can trigger vigoroso inflammation by activating inflammasomes in multiple ways, including reactive oxygen species (ROS), mitochondrial DNA (mtDNA) and cardiolipin. Meanwhile, mtROS-related activation of NF-kB and HIF-1a, mtDNA-induced cGAS-STING pathway, as well as cardiolipin-driven TLRs also contribute a lot during the immune response. Notably, as the center of metabolism, various metabolic pathways and metabolites within mitochondria must also not be ignored in the process. **(B)** SLE is promoted by mitochondrial signaling through numerous mechanisms. 1) mROS not only directly causes severe inflammation and tissue damage by itself but also contributes to NETosis and oxidize mtDNA into oxidized form with enhanced inflammatory capability. However, with the accumulation of mROS, NOX complex-related ROS is inhibited. 2) Both prototype and oxidized form mtDNA can be recognized by TLR9 or NLRP3 to evoke downstream immune signaling. Meanwhile, there is a brunch of pathways for their accumulation. With the activation of TLR7 by anti-Sm/RNP antibody under type I IFN environment, the phosphorylation of TAFM is blocked and persistently covered in the oxidized mtDNA to protect it. And other self-clearance ways are also weakened. The HRES-1/Rab4 enzyme can digest Drp1 to prevent physiological mitophagy, resulting in increased mitochondria mass for indirect mtDNA accumulation. 3) Similarity to mtDNA, cardiolipin can be recognized by TLR4 or NLRP3 to evoke inflammation, but it can also be recognized and bound by anti-phospholipid antibodies, one type of classical autoantibodies of SLE, to form the immune complex and induce subsequent effects. **(C)** RA is promoted by mitochondrial signaling in the following mechanisms. 1) Except for systematically accumulating mROS and correspondingly decreased NOX complex-related ROS and increased oxidized mtDNA, it can directly gather in the synovial joint to cause immensity local damage. 2) mtDNA and its oxidized form can activate cGAS-STING or NLRP3 for inflammation. Meanwhile, failed self-DNA degradation by DNase II and attenuation of PINK1-mediated mitophagy caused by TNF promote this process. 3) Cardiolipin can trigger NLRP3 inflammasome for inflammation.

Mitochondria, as a critical organelle in a cell for energy supply, is now recognized to have comprehensive and sophisticated functions in maintaining homeostasis and also innate immunological responses. The immunological function of mitochondria is of great importance in deciphering the pathogenesis of immune disorders. And compiling studies show the close relationship between mitochondria abnormalities and the pathogenesis of autoimmune diseases (**Figure 1**). In response to immunological stimuli, the stress signal activates respective signaling pathways including TLRs, NLRs, and RLRs, eventually leading to the production of proinflammatory cytokines and augmentation of immune responses. As one of the major sources of host- derived DAMPs, mitochondria generate multiple types of alarmins including mROS, mtDNA, and cardiolipins. These molecules play a pivotal role in initiation and activation of certain immune responses or mediating direct tissue damage. Abnormalities of DAMPs generation of signal transduction of TLRs, NLRs, and RLRs are found in multiple autoimmune diseases. By directly targeting DAMPs or signaling pathways of TLRs, NLRs, and RLRs, people could discover novel treatments for autoimmune diseases. And the attempts to develop therapeutic strategies for SLE and RA by targeting mitochondrial molecules and their respective signaling pathways are summarized in **Table 1**. Mitochondria also controls the metabolism of innate immune cells, which further interferes with their function in innate immunity. The dysregulation of metabolic pathways and metabolites has been linked to several autoimmune diseases. Although several mitochondrial pathways involved in the pathogenesis of autoimmune diseases have been targeted for therapeutic strategies (as shown in **Figure 1**), many remain unexplored. Targeting these unattended pathways with novel agents that modulate metabolic pathways and metabolites could offer potential therapeutic options for autoimmune diseases and should be further investigated.

**Table 1:** Potential therapeutic agents targeting mitochondrial molecules in SLE and RA.

Mitochondrial target	Therapeutic agent	Preclinical or Clinical Findings	References
mROS	mitoQ	SLE: Inhibits MAVS and results in decreased mROS and reduced NETosis	(34, 42, 43)
	mitoTEMPO		
	Drp1/Rab4A	SLE: Rab4A mediated Drp1 depletion diminish mitophagy and accumulation of mitochondria mass	(40, 41)
	polydatin	SLE: Downregulate ROS and NETosis	-44
	resveratrol	RA: Inhibits ROS and MAPK signaling to suppress angiogenesis and alleviate disease progression	-45
	celastrol	RA: Inhibits ROS and NF-κB-NLRP3 inflammasome	-46
	Metformin/NETosis	SLE: reduce mild to moderate disease relapse by inhibiting NET formation	-36
mtDNA	DNase	SLE and RA: Upregulation of mtDNA in SLE and RA patients	(51, 52)
Cardiolipin	Sam50	SLE: Sam50 overexpression reverse cardiolipin externalization and inhibit inflammation	-95
TLRs	Chloroquine	SLE and RA: Blocks TLRs and NF-kB translocation to inhibit pro-inflammatory cytokine production	-127
	Hydroxychloroquine		

*Indirect evidence is not shown in the list.

In general, the actual mechanisms that induce autoimmune disease pathogenesis are complicated and might involve far more molecules and signaling pathways. In the study into such disease, mitochondria with their related molecules, signaling pathways, and metabolic pathways could possibly be a potential key to the question.

## Author contributions

YJ and ZY did the literature review and drafted the manuscript. AY supervised on finishing this review. All authors read and approved the final manuscript for publication.
